# Localization of plasma membrane t-SNAREs syntaxin 2 and 3 in intracellular compartments

**DOI:** 10.1186/1471-2121-6-26

**Published:** 2005-05-19

**Authors:** Arja M Band, Esa Kuismanen

**Affiliations:** 1 Haartman Institute and Molecular and Cancer Biology Program, Biomedicum Helsinki, Haartmaninkatu 8, 00014 University of Helsinki, Finland; 2 Department of Biosciences, Division of Biochemistry, Viikki Biocenter, Viikinkaari 5, 00014 Helsinki, Finland

## Abstract

**Background:**

Membrane fusion requires the formation of a complex between a vesicle protein (v-SNARE) and the target membrane proteins (t-SNAREs). Syntaxin 2 and 3 are t-SNAREs that, according to previous over-expression studies, are predominantly localized at the plasma membrane. In the present study we investigated localization of the endogenous syntaxin 2 and 3.

**Results:**

Endogenous syntaxin 2 and 3 were found in NRK cells in intracellular vesicular structures in addition to regions of the plasma membrane. Treatment of these cells with N-ethylmaleimide (NEM), which is known to inactivate membrane fusion, caused syntaxin 3 to accumulate in the trans-Golgi network and syntaxin 2 in perinuclear membrane vesicles. Kinetic analysis in the presence of NEM indicated that this redistribution of syntaxin 2 and 3 takes place via actin containing structures.

**Conclusion:**

Our data suggest that syntaxin 2 cycles between the plasma membrane and the perinuclear compartment whereas syntaxin 3 cycles between the plasma membrane and the trans-Golgi network. It is possible that this cycling has an important role in the regulation of t-SNARE function.

## Background

Membrane traffic is needed for the synthesis and processing of proteins and lipids as well as the maintenance of the compartmentalization of the cell. Trafficking of intracellular membranes involves the budding of vesicles from the donor membrane and the fusion of vesicles with their respective target membranes. Several proteins are involved in membrane fusion events, including the N-ethylmaleimide (NEM)-sensitive factor (NSF), soluble NSF attachment proteins (SNAPs) and SNAP receptors (SNAREs). SNAREs are a super family of integral membrane proteins characterized by α-helical motif. The SNAREs that are functioning in neuronal exocytosis are best characterized. They include the vesicle SNARE synaptobrevin (also referred to as VAMP, vesicle-associated membrane protein) and the membrane proteins SNAP-25 and syntaxin 1 [1]. The pairing of target SNARE (t-SNARE) with the vesicle SNARE (v-SNARE) (trans complex) pulls the membranes together and this is possibly the driving force in the mixing of the lipid bilayers. SNAREs form bundles which contain four α-helices in a parallel arrangement [2]. In the middle of the hydrophobic bundle, there is a hydrophilic section which either contains three conserved glutamines (Q) or one conserved arginine (R). This led to the classification of SNAREs into Q-SNAREs and R-SNAREs [3]. For instance, SNAP-25 and syntaxins are Q-SNAREs and VAMP is an R-SNARE. Three helices of the helical bundle come from Q-SNAREs and one from an R-SNARE. Syntaxins and VAMP contain one helical SNARE motif but SNAP-25 contains two motifs [2]. The disassembly of the SNARE complexes that are formed is mediated by NSF attachment proteins, SNAPs, and the ATPase activity of NSF [1,4].

A unique set of SNAREs is located in distinct intracellular compartments. Liposome fusion assay has demonstrated that SNAREs do show high specificity in forming complexes with each other [5]. The formation of functional trans complexes was mostly restricted to physiologically relevant SNARE combinations. The specificity of the complex formation resides in the SNARE motifs [6]. However, v-SNAREs are present in both anterograde and retrograde vesicles and therefore other proteins are needed to contribute to the specificity of vesicle targeting [7]. Those proteins include inter alia small Rab GTPases, Sec1 proteins, and complexins [8,9]. Recently it has been reported that the formation of non-cognate SNARE complexes that are non-fusogenic might have a regulatory role. These inhibitory SNAREs have been suggested to increase the specificity of membrane targeting by inhibiting membrane fusion outside their specific compartments [10].

Syntaxins belong to a t-SNARE family of which over a dozen have already been cloned [11]. Over-expression studies have suggested that syntaxin 1, 2, 3, and 4 are located predominantly at the plasma membrane. Syntaxin 1 is mainly expressed in brain tissue and is thought to function specifically in neurotransmitter release, whereas syntaxin 2, 3, and 4 have a wider tissue distribution [12]. We have previously demonstrated that syntaxin 4 is localized, in addition to the plasma membrane, in intracellular vesicular structures as well [13]. These structures co-localized with rab11 staining. Treatment with NEM caused accumulation of syntaxin 4/rab11 positive labelling to actin filaments [13].

In this study, we investigated subcellular localization of endogenous syntaxin 2 and 3 in NRK cells. Similar to syntaxin 4, syntaxin 2 and 3 were found to localize in intracellular vesicular structures in addition to regions of the plasma membrane. In the case of syntaxins 2 and 3, NEM treatment resulted in the accumulation of these proteins in perinuclear membrane vesicles and the trans-Golgi network (TGN), respectively. Kinetic analysis in the presence of NEM suggested that both syntaxin 2 and 3 were redistributed to the perinuclear sites through actin containing structures.

## Results

### Characterization of syntaxin 2 and 3 anti-sera

**Figure 1:**
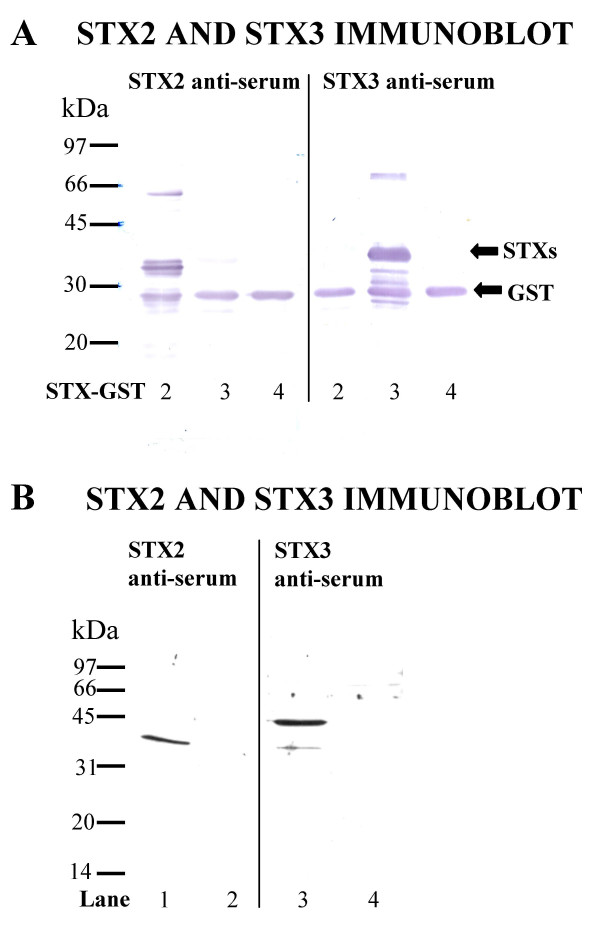
**Characterization of the syntaxin 2 and 3 anti-sera**. (A) Equal amounts of syntaxin 2, 3, and 4 cytosolic domain GST fusion proteins (2 μg) were incubated with thrombin (0.3 U) for two hours to cleave off the GST (27 kDa, indicated with arrow) and analysed by SDS-PAGE, transferred to nitrocellulose filters and probed with syntaxin 2 or 3 anti-serum followed by alkaline phosphatase-conjugated secondary antibodies. (B)The Western blotting of enriched membrane fraction of NRK cells were probed with syntaxin 2 anti-serum (lane 1), syntaxin 2 anti-serum pre-incubated with syntaxin 2-GST protein (lane 2), syntaxin 3 anti-serum (lane 3) and syntaxin3 anti-serum preincubated with syntaxin 3-GST protein (lane 4) as well as horseradish peroxidase conjugated secondary antibodies. Each lane contains 25 μg protein. All the samples were boiled for 3 minutes in the presence of 2% SDS in Laemmli sample buffer.

Previous over-expression studies have suggested that syntaxins 1, 2, 3, and 4 are primarily localized at the plasma membrane [12,14]. However, we have previously found that endogenous syntaxin 4 is localized in rab 11 positive intracellular membranes as well as at the plasma membrane [13]. Therefore in this study we investigated the localization of endogenous syntaxin 2 and 3. Anti-sera against syntaxin 2 and 3 were raised by immunization of rabbits with the amino terminal cytosolic domains of rat syntaxin 2 and 3. Since syntaxins 2, 3 and 4 are highly homologous to each other [12], we first determined the specificity of the anti-sera. Both syntaxin 2 and 3 anti-sera specifically recognized only their respective antigens and did not cross-react with the cytosolic domain of other syntaxins on Western blotts (Fig. 1A). Enriched membrane fraction of NRK cells was resolved with SDS-PAGE and blotted with syntaxin 2 or 3 anti-serum. Syntaxin 2 anti-serum blotted a band migrating close to the calculated molecular weight of syntaxin 2 monomer 34 kDa (Fig. 1B, lane 1). This band was abolished with pre-incubation of the syntaxin 2 anti-serum with syntaxin 2 GST-protein (Fig. 1B, lane 2). When the membrane fraction was blotted with syntaxin 3 anti-serum two bands appeared migrating with the apparent molecular masses of approximately 33 kDa and 40 kDa. Both of these bands were abolished by the pre-incubation of the syntaxin 3 anti-serum with recombinant syntaxin 3. These bands most likely represent two different forms of monomeric syntaxin 3. This conclusion is supported by previous observations, which indicate that mouse brain contains four different forms of syntaxin 3 and two forms of syntaxin 1 that migrate at different molecular weights [15,16]. Our syntaxin 3 anti-serum has also been previously shown to blot syntaxin 3 at the apparent molecular weight of approximately 40 kDa in Caco-2 cell extract [17].

### Endogenous syntaxin 2 and 3 were found to be localized in intracellular compartments

**Figure 2:**
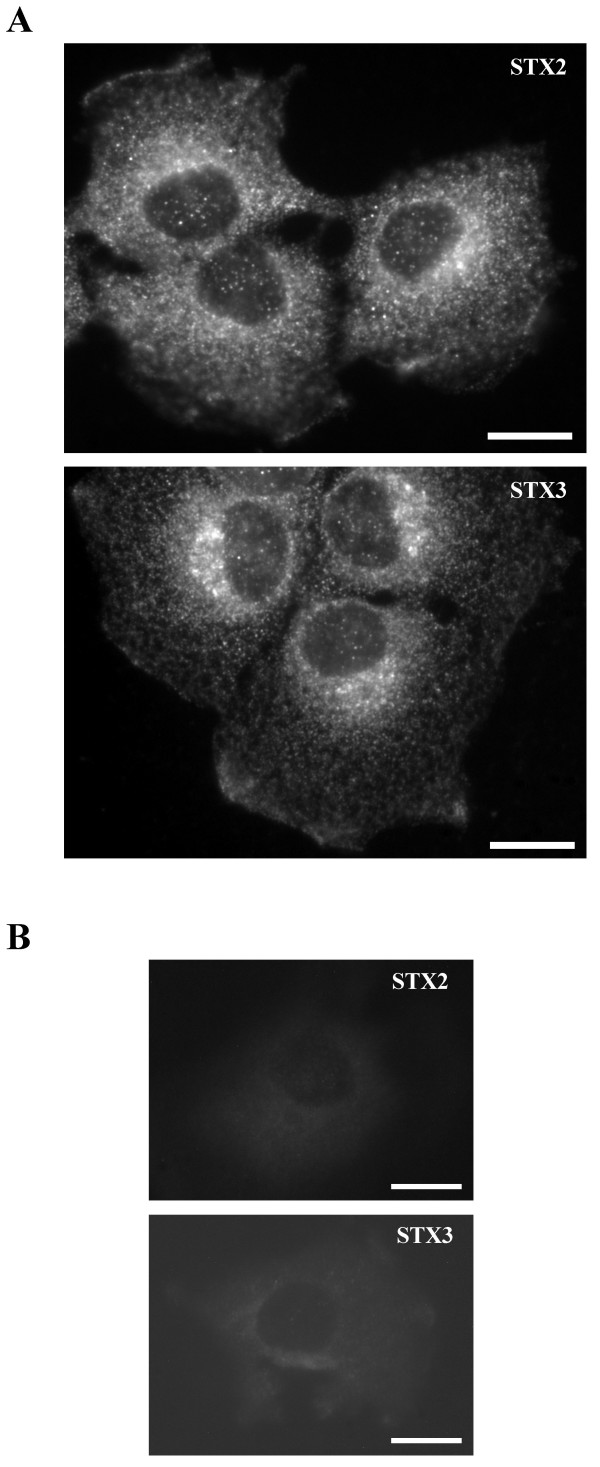
**Syntaxin 2 and 3 anti-sera stained intracellular vesicular structures and regions of the plasma membrane in NRK cells**. The cells were fixed with 0.0 8 M lysine-0.01 M periodate-2% paraformaldehyde and permeabilized with 0.05% saponin. Syntaxin 2 and 3 were visualized using syntaxin 2 and 3 anti-sera and LRSC-conjugated goat anti-rabbit IgG. Conventional fluorescence images were viewed using an Olympus AX70 fluorescence microscope. Bars,10 μm.

**Figure 3:**
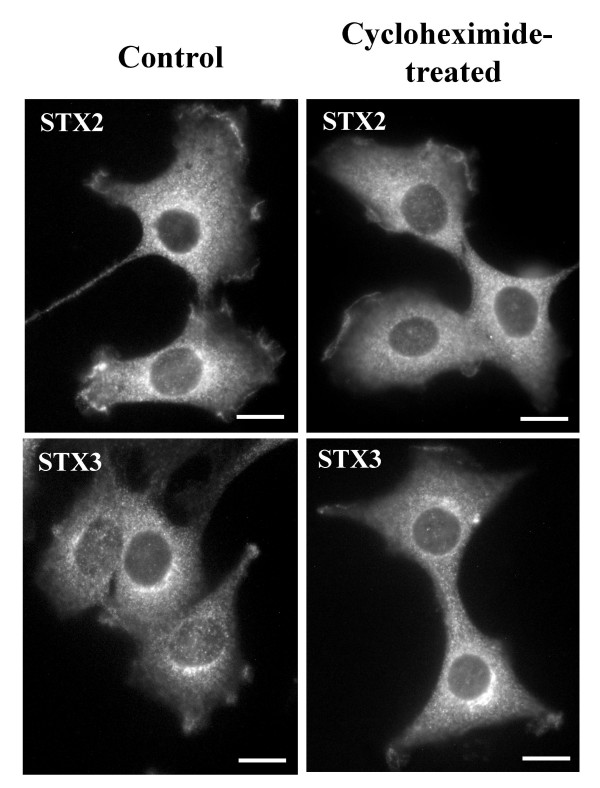
**The intracellular syntaxin 2 or 3 antibody labelled structures do not represent newly synthesized syntaxin 2 or 3 proteins**. The NRK cells were treated with 50 μg/ml cycloheximide for two hours. The cells were fixed with 0.08 M lysine-0.01 M periodate-2% paraformaldehyde and permeabilized with 0.05% saponin. Syntaxin 2 and 3 were visualized using syntaxin 2 and 3 anti-sera and LRSC-conjugated goat anti-rabbit IgG. Conventional fluorescence images were viewed using an Olympus AX70 fluorescence microscope. Bars,10 μm.

Syntaxin 2 and 3 anti-sera stained intracellular vesicular structures and regions of the plasma membrane in NRK cells (Fig. 2A). These stainings can be totally blocked with recombinant syntaxin 2 and 3 proteins (Fig. 2B). Since these syntaxins are thought to be located at the plasma membrane it was considered possible that the syntaxin 2 and 3 positive intracellular vesicles were constitutive exocytic vesicles carrying syntaxins as cargo. To study whether this is the case, we treated cells with cycloheximide for two hours to deplete newly synthesized proteins from the biosynthetic pathway membranes (Fig. 3). This treatment did not abolish the intracellular vesicular labelling, demonstrating that the syntaxin 2 and 3 positive membranes did not represent the newly synthesized syntaxins on their way to the plasma membrane.

### In the presence of NEM syntaxin 2 accumulates in perinuclear vesicles and syntaxin 3 in the TGN

**Figure 4:**
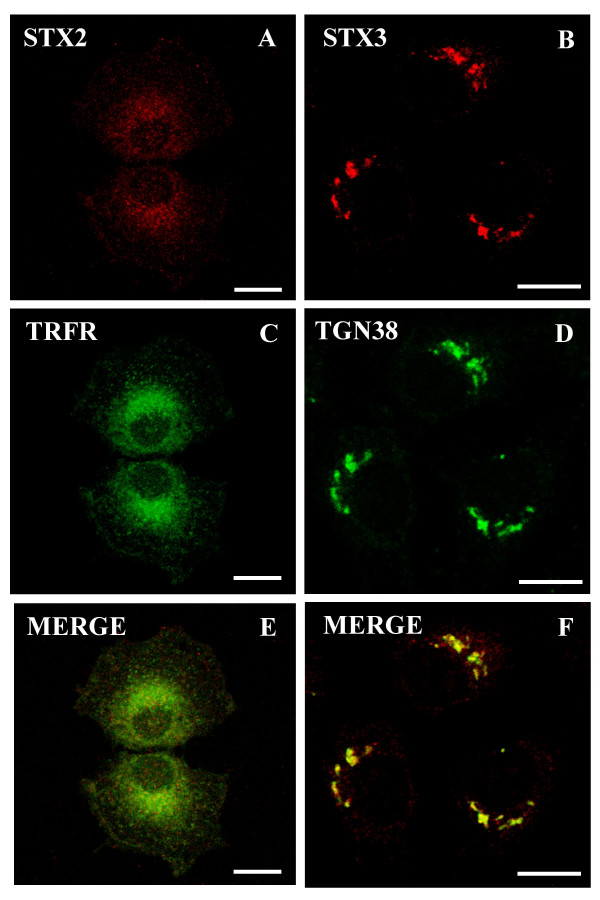
**Syntaxin 2 localized in perinuclear membrane vesicles and syntaxin 3 localized in the TGN in NEM treated NRK cells**. The NRK cells were incubated in the presence of 1 mM NEM for 15 minutes and then further incubated for two hours. Both syntaxin 2 and 3 accumulated into intracellular compartments in the presence of NEM (A,B). The cells were double stained with syntaxin 2 (A) or syntaxin 3 (B) anti-serum and LRSC-conjugated goat anti-rabbit IgG as well as antibodies against transferrin receptor (Ox26) (C) and TGN38 (D) mouse monoclonal antibodies and FITC-conjugated goat anti-mouse IgG. The yellow colour in merged images (E and F) reveals the co-localization. Confocal fluorescence images were viewed using a Leica SP1 microscope system. Bars,10 μm.

**Figure 5:**
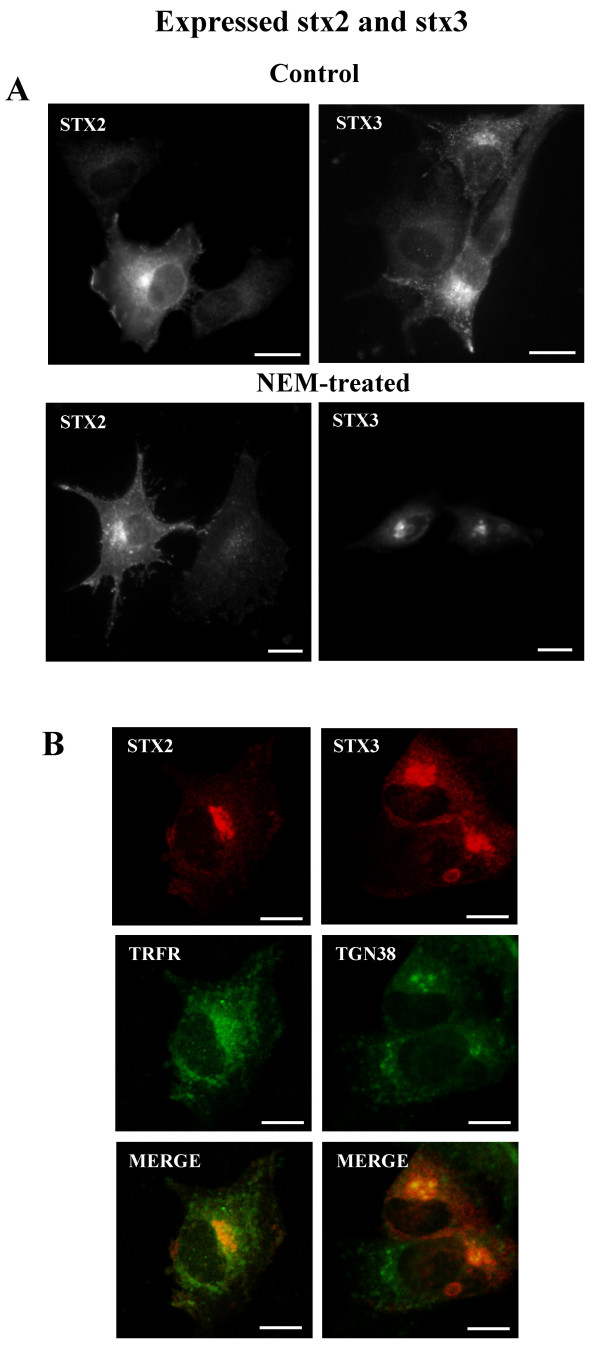
**The effect of NEM on the localization of expressed syntaxin 2 and 3 in NRK cells**. Syntaxin 2 and 3 were expressed in NRK cells and stained with syntaxin 2 or 3 antiserum. (A) Control cells show localization of expressed syntaxins both at the plasma membrane and in intracellular sites. The control cells were treated with 50 μg/ml cycloheximide (controls). Also expressed syntaxin 2 and 3 accumulated into intracellular sites in the presence of NEM. (B) NEM-treated cells were double stained using syntaxin 2 or syntaxin 3 anti-serum and monoclonal antibodies against transferrin receptor (Ox26) or TGN38 as in Fig. 4. The yellow colour in merged images reveals the co-localization. Exposure in the pictures was adjusted so that only expressed syntaxins can be seen. Conventional fluorescence images were viewed using an Olympus AX70 fluorescence microscope (A). Confocal fluorescence images were viewed using a Leica SP1 microscope system (B). Bars, 10 μm.

Next we investigated how the inhibition of the SNARE complex disassembly with NEM affects the localization of syntaxin 2 and 3. NEM is a sulphydryl alkylating reagent, which has been reported to inactivate the membrane fusion component NSF [18]. We have previously shown that NEM treatment stopped membrane transport from the TGN to the plasma membrane [19]. We have also found that NEM treatment caused the majority of the syntaxin 4/rab 11 positive staining to move from intracellular vesicular structures to actin filaments [13]. Since NEM treatment resulted in a dramatic redistribution of syntaxin 4, we studied the localization of syntaxin 2 and syntaxin 3 in similar experiments. Interestingly, NEM treatment affected the localization of syntaxin 2 and syntaxin 3 differently from syntaxin 4. In the presence of NEM endogenous syntaxin 2 and 3 accumulated in perinuclear membrane structures and very little staining of these syntaxins can be observed in vesicles or at the plasma membrane (Fig. 4). We used markers to study the site of accumulation of endogenous syntaxins. In the presence of NEM endogenous syntaxin 2 partly co-localized with transferrin receptor and did not co-localize with a late endosome marker, lyso-bis-phosphatidic acid (not shown) (Fig. 4). Some endosomal association for syntaxin 2A and 2B has been reported previously as well [20]. Endogenous syntaxin 2 was found to be partly co-localized with v-SNARE, cellubrevin in the perinuclear compartment. In the presence of NEM endogenous syntaxin 3 accumulated in perinuclear elements which co-localized with the TGN marker, TGN38 (Fig. 4). Similar redistribution of syntaxin 2 and 3 in the presence of NEM can also be observed when syntaxin 2 or 3 is transiently expressed from the cDNA in NRK cells (Fig 5). In control cells the syntaxins were seen both at the plasma membrane and in intracellular sites (Fig. 5A). After NEM- treatment the labelling of expressed syntaxins at the plasma membrane was greatly reduced and the syntaxins were seen accumulated in intracellular perinuclear elements similar to the endogenous syntaxin 2 or 3 (Fig. 5B). The different distribution of syntaxin 2, 3 and 4 in the presence of NEM suggests that these syntaxins are not only present at the plasma membrane but are also cycling between the plasma membrane and different intracellular compartments of the cell. According to the present results syntaxin 3 might be the SNARE responsible for the fusion of the exocytic carriers to the plasma membrane in NRK cells. This is supported by the previous observation that syntaxin 3 is present in zymogen granules of pancreatic acinar cells [21,22].

### Redistribution of syntaxin 2 and 3 takes place via actin containing structures

**Figure 6:**
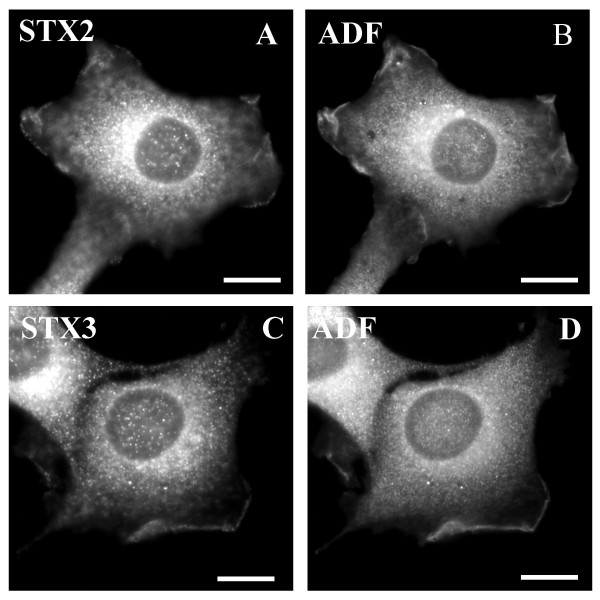
**Syntaxin 2 and 3 staining coincides with actin at cortical regions**. The NRK cells were fixed, permeabilized and then double stained with syntaxin 2 (A) or 3 (C) anti-serum, LRSC-conjugated goat anti-rabbit IgG as well as guinea pig anti-α-ADF (B,D) and FITC-conjugated donkey anti-guinea pig IgG. Conventional fluorescence images were viewed using an Olympus AX70 fluorescence microscope. Bars, 10 μm.

**Figure 7:**
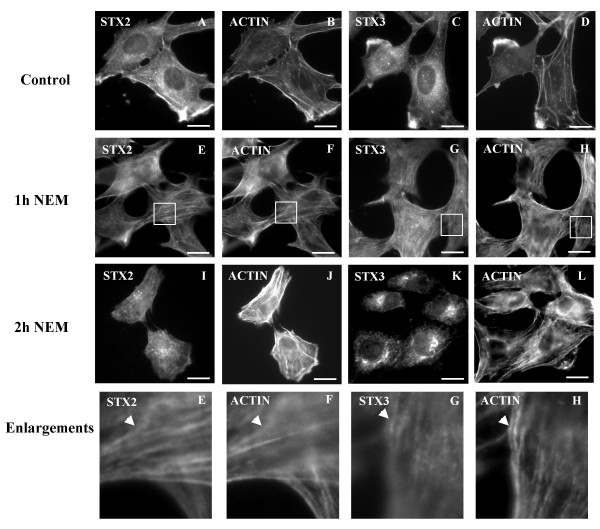
**Syntaxin 2 and 3 can be transported to their intracellular sites along actin filaments**. The NRK cells were incubated in the presence of 1 mM NEM for 15 minutes and then further incubated for one or two hours. Syntaxin 2 and 3 were visualized using syntaxin 2 and 3 anti-sera and LRSC-conjugated goat anti-rabbit IgG; actin filaments were stained with Oregon Green phalloidin. Enlargements of the boxed areas are shown below. The arrows show the colocalization of syntaxin 2 and 3 staining with actin staining in filament-like structures after one hour of NEM-treatment. Conventional fluorescence images were viewed using an Olympus AX70 fluorescence microscope. Bars, 10 μm.

We have previously observed that in the presence of NEM syntaxin 4 accumulates on actin filaments and is directly associated with actin. In contrast to this, syntaxin 2 or 3 did not cosediment with polymerizing actin filaments suggesting that these syntaxins are not directly associated with actin [13]. However, syntaxin 2 and 3 positive vesicles were not redistributed when microtubules were depolymerised with cold treatment and repolymerization was prevented with nocodazole (not shown). Therefore we investigated the possible involvement of actin filaments in the transport of syntaxin 2 and 3 vesicles from the plasma membrane to the intracellular sites. Little is known about the role of actin cytoskeleton in membrane transport in animal cells. However, recently some evidence has emerged suggesting that in addition to microtubules actin cytoskeleton is involved in membrane/organelle transport. The actin cytoskeleton promotes internalization of ligands and vesicle trafficking along the endosomal pathway as well as the movement of membrane elements such as lysosomes [23-25]. We used an actin monomer binding protein cofilin/actin depolymerising factor, ADF, as a marker of dynamic region of actin cytoskeleton [26]. Both syntaxin 2 and 3 co-localized with ADF at the plasma membrane (Fig. 6). Also another actin marker, Oregon Green phalloidin, co-localized with syntaxin 2 and 3 in actin rich areas at the plasma membrane (Fig 7A–7D). When we studied the time course of the localization of syntyaxin 2 and 3 we observed that both syntaxin 2 and 3 were associated in filament-like structures after one hour's treatment with NEM (Fig. 7E–7H). These structures co-localized with actin. After two hours' treatment with NEM syntaxin 2 and 3 accumulated at their perinuclear destinations without actin association (Fig 7I–7L). This result with syntaxin 2 and 3 is in contrast to our previous observation which indicated that syntaxin 4 stays associated with actin filaments even after two hours of NEM treatment [13].

**Figure 8:**
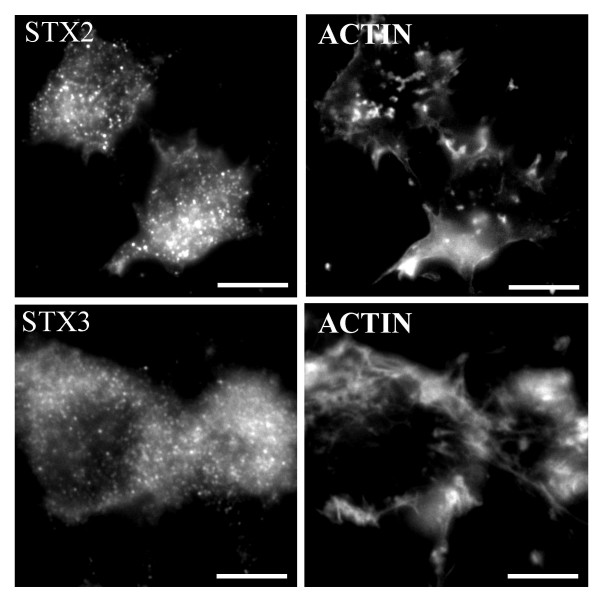
**Syntaxin 2 or 3 staining does not accumulate into actin containing aggregates after depolymerization of actin filament**. The NRK cells were treated with 10 μM cytochalasin D for 30 minutes to disrupt the actin filaments. The cells were fixed, permeabilized and then stained with syntaxin 2 or 3 anti-serum and LRSC-conjugated goat anti-rabbit IgG as well as Oregon Green phalloidin. Conventional fluorescence images were viewed using an Olympus AX70 fluorescence microscope. Bars, 10 μm.

We have previously observed that the disruption of the actin cytoskeleton with cytochalasin D accumulated syntaxin 4 into actin containing aggregates [13]. However, when we investigated the effect of cytochalasin D on syntaxin 2 and 3 labelling no accumulation into actin aggregates was observed (Fig. 8). Both syntaxin 2 and 3 stayed in vesicular structures.

## Discussion

In the present study, we have shown that endogenous plasma membrane t-SNAREs syntaxin 2 and 3 are not exclusively localized at the plasma membrane. In addition to the plasma membrane localization, syntaxin 2 and 3 were found to localize in intracellular membrane compartments as well. Treatment with NEM caused syntaxin 2 to accumulate in perinuclear vesicular structures which partly co-localized with the transferrin receptor whereas syntaxin 3 accumulated in the TGN. It is therefore possible that syntaxin 2 might cycle between the plasma membrane and the perinuclear membrane vesicles, and syntaxin 3 between the plasma membrane and the TGN in NRK cells. Kinetic analysis suggested that actin cytoskeleton is involved in recycling of syntaxin 2 and 3 to the perinuclear sites.

Our immunofluorescence microscopy studies indicate that endogenous syntaxin 2, 3 and 4 are located only in short sections of the plasma membrane and they are not dispersed all over of the plasma membrane. Syntaxin 2, 3 and 4 co-localize with ADF, a marker for highly dynamic regions of the actin cytoskeleton. This indicates that each of these syntaxins is present in the same section of the plasma membrane and these syntaxins cycle between this active section of the plasma membrane and different intracellular sites. In previous reports it has been suggested that syntaxin 2 is present both at the apical and basolateral portion of the plasma membrane and syntaxin 3 at the apical portion of the plasma membrane in polarized cells [21,27,28]. Therefore it is possible that syntaxins are targeted to the active sections of the plasma membrane in non-polarized cells as well. The cycling of syntaxins would make it possible to ensure that all the components of the fusion machinery are correctly targeted to an active site in an active form.

We have previously observed that syntaxin 4 is directly associated with actin as examined by using a cosedimentation assay. Syntaxin 2 and 3, on the other hand, are not directly associated with actin [13]. Kinetic studies indicated that syntaxin 2 and 3 transiently co-localize with actin containing filaments when transported to the perinuclear sites, whereas syntaxin 4 accumulates into actin filament like structures in the presence of NEM [13]. These actin bundles are morphologically distinct from stress fibers and may result from altered assembly kinetics of actin filaments [29]. Disassembly of actin fibers with cytochalasin D causes the accumulation of actin containing aggregates. Syntaxin 4 accumulates with actin into these aggregates [13]. In contrast, syntaxin 2 and 3 stay in vesicular structures in the presence of cytochalasin D. This suggests that syntaxin 2 and 3 are not as tightly attached to actin as syntaxin 4. Interestingly, depolarization of Madin-Darby canine kidney epithelial cells (MDCK) caused relocalization of the apical and basolateral plasma membrane to functional apical and basolateral vacuoles, respectively. These vacuoles are associated with actin cytoskeleton [28].

In a few previous investigations endogenous intracellular syntaxin 2 or 3 labelling has also been observed. In those studies it has been suggested that syntaxin 2 and 3 have another role in intracellular membrane fusion processes besides the fusion process at the plasma membrane. It has been reported that syntaxin 2, 3 and 4 are present in phagosomal membranes and suggested that they are involved in phagosomal maturation [30]. Similarly, syntaxin 3 was found in granular membranes of the zymogenic cells and a role of granule-granule fusion was suggested [22]. Also in over-expression studies intracellular syntaxin labelling has been observed and it has been thought to be caused by over-expression or mislocalization [12]. We used lysine-periodate-paraformaldehyde fixation and saponin permeabilization in our studies to preserve the intracellular vesicular structures and therefore made them more visible than if a standard paraformaldehyde fixation had been used.

## Conclusion

The present study clearly indicates that syntaxin 2 and 3 are not solely localized at the plasma membrane but are also present in intracellular compartments and that they may cycle between these compartments and the plasma membrane through actin containing structures. This cycling of syntaxins is likely to have an important role in the regulation of t-SNARE function.

## Methods

### Materials

Mouse monoclonal anti-rat TGN38 antibody was a gift from Dr. G. Banting (University of Bristol, Bristol, UK.) and mouse monoclonal anti-rat transferrin receptor Ox26 hybridoma cell line was obtained from Peninsula Laboratories, Inc. (San Carlos, CA). Actin monomer binding protein cofilin/actin depolymerizing factor (ADF) guinea pig affinity purified anti-serum [31] was a gift from Dr. P. Lappalainen (Institute of Biothechnology, Helsinki, Finland). Plasmids encoding full length mouse syntaxin 2A and rat syntaxin 3A cDNAs in pBK-CMV vectors (Stratagen, La Jolla, CA USA) were gifts from Dr. V. Olkkonen (National Public Health Institute, Helsinki, Finland). Lissamine rhodamine (LRSC)-conjugated, Rhodamine Red™-X-conjugated and fluorescein (FITC)-conjugated secondary antibodies were purchased from Jackson Immuno Research (West Grove, PA, USA). All other reagents were of analytical grade and were obtained from commercial sources.

### Production of fusion proteins and antibodies

The cytosolic domain of mouse syntaxin 2 (1–265), rat syntaxin 3 (1–263) and rat syntaxin 4 (1–272) in pGEX 2T vectors and syntaxin 2 and 3 antisera were gifts from Dr. V. Olkkonen (National Public Health Institute, Helsinki, Finland). The production and the purification of GST fusion proteins were performed according to the manufacturer's instructions (Amersham Pharmacia Biotech AB, Uppsala, Sweden). The anti-serum for syntaxin 4-GST proteins was produced in New Zealand White rabbits.

### Cell culture

Normal rat kidney (NRK) cells were grown at 37°C in 5% CO_2 _in DME supplemented with 2 mM L-glutamine, 100 U of penicillin, 10 mg/ml of streptomycin, and 10% (v/v) foetal calf serum (Biological Industries, Beit Haemek, Israel). NEM-treatment was performed by incubating cells in 1 mM NEM for 15 minutes and then further incubating in serum free DME medium.

### Western blotting

The samples were dissolved into SDS-Laemmli buffer, separated in SDS-PAGE, and transferred to Hybond ECL nitrocellulose membrane (Amersham Pharmacia Biotech AB, Uppsala, Sweden). Nonspecific binding of antibodies was blocked with 5% fat-free milk in TBST buffer (0.15 M NaCl, 0.05% Tween 20, 10 mM Tris-HCl pH 8.0). Secondary antibodies were conjugated with either alkaline phosphatase (Sigma, St. Louis, MO, USA) or horseradish peroxidase (Bio-Rad Laboratories, Hercules, California, USA). Alkaline phosphatase and ECL reactions were performed according to the manufacturer's instructions (Promega, Madison, WI, USA and Amersham Pharmacia Biotech AB, Uppsala, Sweden, respectively).

### The preparation of membrane fraction

NRK cells were grown as confluent monolayers on 10 cm dishes. The cells were washed with hypotonic swelling buffer (10 mM KCl, 5 mM MgCl_2_, 10 mM Tris-HCl pH 7.2) and scraped in transport medium (115 mM Kacetate, 3.5 mM MgCl_2_, 1 mM EGTA, 1 mM DTT, 0.1 mM PMSF, 25 mM Hepes-KOH pH 7.4) in the presence of 1 mM phenylmethylsulfonyl fluoride, 10 μg/ml leupeptin and 2 μg/ml pepstatin A. The cells were then distrupted by repeated passage through a 23-gauge needle. The homogenate was first centrifuged at 5 000 g for 20 minutes and then the supernatant was further centrifuged at 100 000 g for one hour to precipitate membranes.

### Immunocytochemistry

NRK cells were grown as confluent monolayers on coverslips in DME. The cells were fixed with 0.08 M lysine-0.01 M periodate-2% paraformaldehyde [32] and permeabilized with 0.05 % saponin to maintain vesicular structures. Conventional fluorescence images were viewed using an Olympus AX70 fluorescence microscope with a SenSys CCD camera (Photometrics, Ltd., Munich, Germany). Images were converted using the Image-Pro Plus version 3.0 software (Media Cybernetics, Silver Spring, MD, USA). Confocal images were recorded using a laser scanning Leica SP1 confocal microscope. One layer was superimposed in the image.

## Abbreviations

ADF, actin monomer binding protein cofilin/actin depolymerizing factor; NEM, N-ethylmaleimide; NRK, normal rat kidney; NSF, N-ethylmaleimide-sensitive fusion factor; SNAP, soluble NSF attachment proteins; SNARE, SNAP receptor; stx, syntaxin; TGN, trans-Golgi network; VAMP, vesicle-associated membrane protein

## Authors' contributions

AMB carried out all the experiments and wrote the manuscript. EK gave advice on experiments and edited the manuscript. Both authors read and approved the final manuscript.
